# Desmosomal Molecules In and Out of Adhering Junctions: Normal and Diseased States of Epidermal, Cardiac and Mesenchymally Derived Cells

**DOI:** 10.1155/2010/139167

**Published:** 2010-06-30

**Authors:** Sebastian Pieperhoff, Mareike Barth, Steffen Rickelt, Werner W. Franke

**Affiliations:** ^1^Helmholtz Group for Cell Biology, German Cancer Research Center, Im Neuenheimer Feld 581, 69120 Heidelberg, Germany; ^2^Department of Zoology and Faculty of Land and Food Systems, University of British Columbia, 2357 Main Mall, Vancouver, BC, Canada V6T 1Z4; ^3^Progen Biotechnik GmbH, Maaßstraße 30, 69123 Heidelberg, Germany

## Abstract

Current cell biology textbooks mention only two kinds of cell-to-cell adhering junctions coated with the cytoplasmic plaques: the desmosomes (*maculae adhaerentes*), anchoring intermediate-sized filaments (IFs), and the actin microfilament-anchoring adherens junctions (AJs), including both punctate (*puncta adhaerentia*) and elongate (*fasciae adhaerentes*) structures. In addition, however, a series of other junction types has been identified and characterized which contain desmosomal molecules but do not fit the definition of desmosomes. Of these special cell-cell junctions containing desmosomal glycoproteins or proteins we review the composite junctions (*areae compositae*) connecting the cardiomyocytes of mature mammalian hearts and their importance in relation to human arrhythmogenic cardiomyopathies. We also emphasize the various plakophilin-2-positive plaques in AJs (*coniunctiones adhaerentes*) connecting proliferatively active mesenchymally-derived cells, including interstitial cells of the heart and several soft tissue tumor cell types. Moreover, desmoplakin has also been recognized as a constituent of the plaques of the *complexus adhaerentes* connecting certain lymphatic endothelial cells. Finally, we emphasize the occurrence of the desmosomal transmembrane glycoprotein, desmoglein Dsg2, out of the context of any junction as dispersed cell surface molecules in certain types of melanoma cells and melanocytes. This broadening of our knowledge on the diversity of AJ structures indicates that it may still be too premature to close the textbook chapters on cell-cell junctions.

## 1. Introduction

Typicaldesmosomes (*maculae adhaerentes*) are cell-cell junctions connecting cells of epithelial, meningothelial,and myocardial nature or malignantly transformed cells derived therefrom. Over the past two decades the molecular and ultrastructuralorganization of diverse cell-cell-connecting plasma membrane structures has been elucidated and two novel categories and architectonic principles have been recognized: desmosomal molecules as functionally important components of special non-desmosomal junctions and the existence of certain desmosomal molecules in a non-junction-bound form.

## 2. The Desmosomes of Stratified and Other Epithelia

**Figure 1:**
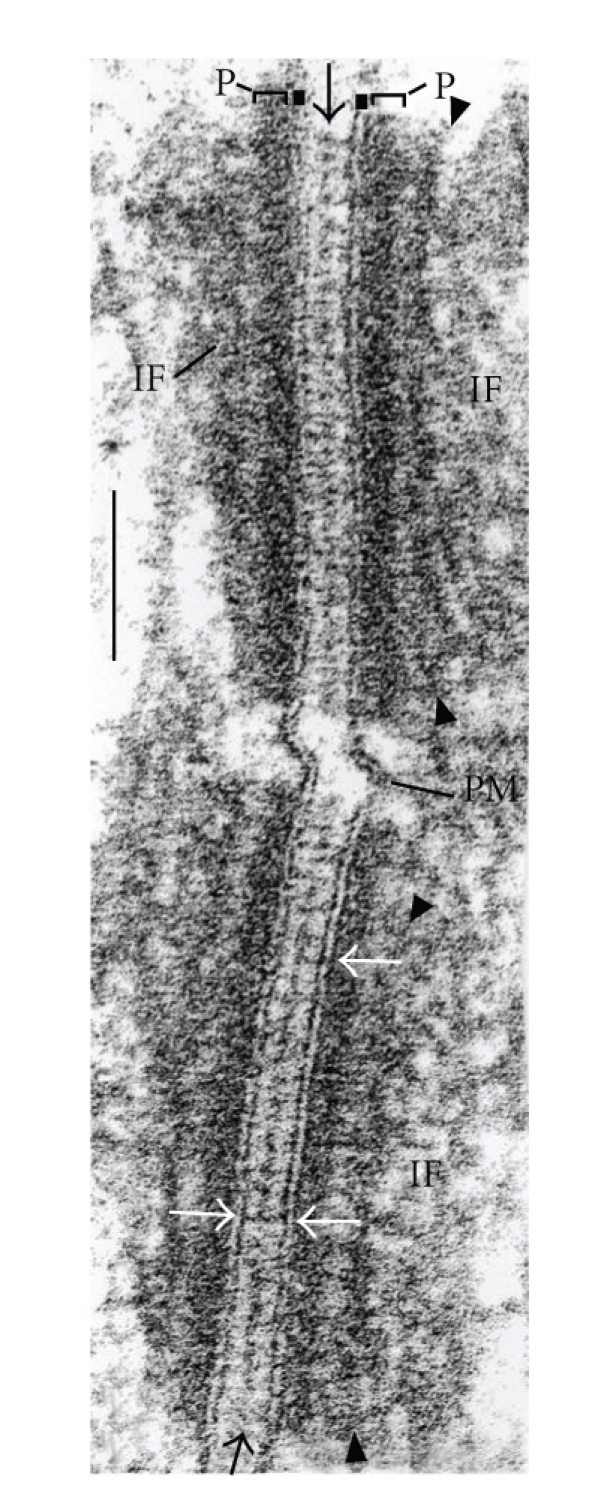
High-magnification electron micrograph of two cross sectioned desmosomal structures connecting *stratum spinosum* cells of human fetal (20 wk) foot-sole epidermis. Brackets and label “P”: cytoplasmic dense plaque; black arrows: midline structure; white arrows: trilaminar “unit membrane” structure of the plasma membrane; arrowheads (top and bottom): secondary dense layer of the plaque; IF: intermediate-sized filaments (for further details see [7]); PM: plasma membrane. Bar: 0.1 *μ*m.

The ultrastructural organization and the high lateral packing density of desmosomes in the epidermis or other multistratified vertebrate epithelia is best seen in the electron microscope (e.g., Figure 1), and the corresponding protein compositions of the various desmosomal subtypes in the specific strata have been determined by biochemical analyses and immunolocalization techniques [1–7].

**Figure 2:**
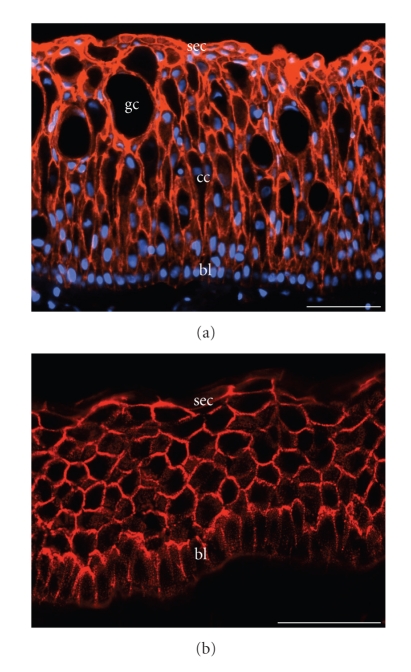
Immunofluorescence microscopy of cryostat sections through fish and amphibian skin, showing the localization of desmosomal components in all layers of the epidermis. (a) Immunofluorescence labeling of plakoglobin (red), in addition to nuclear DAPI staining (blue), in a section through the paraffin-embedded skin of an eel (*Anguilla anguilla*). The section is shown after antigen retrieval and immunoreaction (for methods see [8]) using a monoclonal mouse plakoglobin antibody (mAb PG 5.1; Progen Biotechnik, Heidelberg, Germany). Note the distinct basal layer (*stratum basale*) of the epidermis and the relatively large club shaped cells (“club cells”) as well as the mucous goblet cells and the dense-packed apical cell layers. (b) Immunofluorescence labeling of desmoplakin on a cryostat section through the skin of the frog, *Rana pipiens*, using mAb DP447 (Progen Biotechnik). Note the continuous pattern of very closely spaced, finely punctate staining of the epidermal desmosomes. Bl: basal layer; cc: club cells; gc: goblet cells; sec: superficial epithelial cells (for histological terminology see [8]). Bar in (a): 100 *μ*m; bar in (b): 50 *μ*m.

 In most stratified epithelia, epidermis included, the desmosome packing density is so high that more than half of the entire cell-cell membrane contact area is represented by desmosomal structures. Using immunohistochemical techniques with desmosomal markers this high packing density is directly demonstrable not only for all layers of the highly cornified mammalian epidermis but also in the skin of lower vertebrates such as fishes and amphibia (Figure 2(a) shows the immunolocalization of plakoglobin on a section of fixed, paraffin-embedded skin of the eel, *Anguilla anguilla*; Figure 2(b) shows desmoplakin immunostaining on the skin of the frog, *Rana pipiens*; for details see [8]). Clearly, in stratified epithelia the desmosomal constituents are, together with the keratin filaments, among the most frequent proteins. Moreover, the molecular composition of the epidermal desmosomes—and those of similar multistratified epithelia—has been found to show fundamental strata- specific differences, notably with respect to the desmogleins, Dsg1-4, the desmocollins, Dsc1-3, and the plaque proteins plakophilins, Pkp1-3 (Table 1 and [4–6, 9]). 

 The stratum-specific molecular ensembles, specifically the Dsg and Dsc glycoproteins, are also of marked importance with respect to the pathogenesis of autoimmune skin diseases of the pemphigus type which show a more or less direct correlation with the specific desmosomal glycoprotein complement of the affected layer (see, e.g., [4, 6, 9, 24–28]). These autoimmune diseases are specifically dealt within other articles of this issue. Another aspect of the desmosomal arrays in the epidermis and other stratified epithelia is their frequent—in some areas almost regular—punctuation by very small “sandwich junctions” (*iuncturae structae*) containing the four times-membrane-spanning tight junction hallmark protein, occludin [29, 30].

## 3. The Composite Junctions of the Intercalated Disks (IDs) Connecting Mammalian Cardiomyocytes

**Figure 3:**
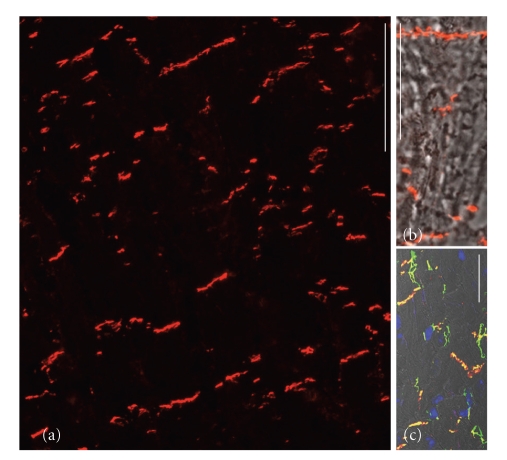
Micrographs showing the immunolocalization of the desmosomal plaque protein, desmoplakin, on cryostat sections through the murine myocardium. (a) Immunofluorescence micrograph showing the localization of desmoplakin (red) in all composite junction structures of the intercalated disks (IDs). (b) Immunofluorescence micrograph showing desmoplakin (red) labeling of the composite junctions on the background of phase-contrast optics. (c) Merged image showing plakophilin-2 (red), *β*-catenin (green) and nuclear DAPI-staining (blue), on an interference contrast microscopy background. Composite junctions (CJs) are characterized by their yellow merge colour whereas the *zonulae adhaerentes* and other AJs of the capillary endothelial cell layers here show only *β*-catenin-positivity. Bar in (a): 100 *μ*m; bar in (b): 50 *μ*m; bar in (c): 25 *μ*m.

**Figure 4:**
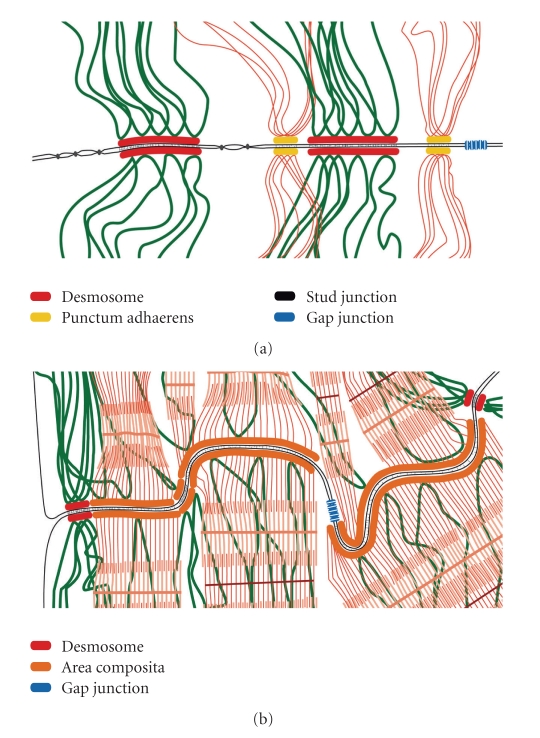
Schematic comparison of the different principles of organization of cell-cell junctions containing desmosomal proteins in mammalian epidermal and myocardiac tissue. (a) Junction organizations between epidermal keratinocytes as observed in the *stratum spinosum*. Note the densely spaced desmosomes (red plaques), which anchor bundles of intermediate sized filaments containing keratins (dark-green). Smaller adherens junctions (*puncta adhaerentia*, yellow plaques) anchor actin-microfilament bundles (red filaments). Furthermore, special types of tight junction-like structures, the small “stud junctions” (black dots), and channel-like connexin paracrystals (gap junctions, blue) are also generally found. For details see [29, 30]. (b) The *area composita* (composite junction, orange) structures of intercalated disks (ID) connecting cardiomyocytes of an adult mammalian heart. This amalgamated type of adhering junction is characterized by a mixture of typical desmosomal and AJ molecules. This kind of composite junction is the predominating adhering junction structure in the ID of the adult mammalian heart.

**Figure 5:**
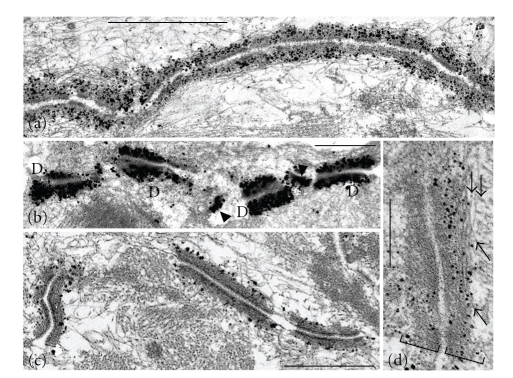
Immunoelectron microscopy of ultrathin sections through bovine myocardium (a) and Purkinje fibers ((b)–(d)) using antibodies against desmoplakin. (a) Immunogold labeling of a typical extended *area composita* structure in an intercalated disk (ID) of adult cardiomyocytes. (b) By contrast, note the relatively small desmoplakin-positive junctions that connect cells of the Purkinje fiber conductive system ((d), desmosomes; arrowheads denote some particularly small desmosome-like junction structures with asymmetric labeling). The desmoplakin-rich plaques of such junctions are very intensely labeled. (c) The morphology and the relatively close packing of the major type of junctions are similar to those of the *area composita* structures of adult mammalian cardiomyocytes. (d) High-magnification immunoelectron micrograph of a composite junction. Note the very close near parallel association of intermediate-sized filaments (some are denoted by arrows) with junctional plaques. Bars in (a) and (b): 1 *μ*m; bar in (c): 0.25 *μ*m.

In mammals the development of the ID junctional system connecting cardiomyocytes does not stop at birth but continues postnatally [15, 31, 32]. In particular the two types of adhering junctions originally distinguishable show further polar translocation, accumulation in the ID region and fusions of the desmosomal and the *fascia adhaerens *components, accompanied by an increasing amalgamation of the two kinds of molecular ensembles into the new mixed category of *area composita *(AC) structures (Figures 3(a)–3(c), 4(b), and 5(a); for details of molecular localizations and biochemical analyses see [14, 15, 33]; for non-mammalian species see however [8, 34]). As a typical result rather extended AC structures are seen which combine compositional and ultrastructural properties of desmosomes with those of AJs (Figures 3 and 4(b)). In addition, the “mixed AC ensembles” of the ID (Table 1; Figure 4(b)) include a series of additional proteins such as *α*-T-catenin [16] and the recently identified plaque protein called “myozap” [17]. The special organizational importance of certain *armadillo*-type proteins, in particular plakoglobin and plakophilin-2, for the entire ID contact of cardiomyocytes has also been demonstrated for early stages of murine heart formation in the absence of the genes encoding these proteins [35, 36] and in siRNA-downregulation experiments ([37–39], for related experiments see also [40, 41]).

The physiological and medical importance of desmosomal molecules in the composite junctions of the myocardiac IDs is most impressively demonstrated by the recent avalanche of publications that specific mutations in genes encoding desmosomal proteins can result in arrhythmogenic cardiomyopathies (ARVC/D), mostly in the right ventricle but left ventricle damages have also been reported (Table 2). Here the gene encoding plakophilin-2 appears to be especially vulnerable as defects in this gene alone seem to account for about two thirds of the cases. This category of ARVC/D-based diseases and “sudden death” events resulting from altered desmosomal proteins also includes complex hereditary syndromes such as combinations of dermatological disorders (“woolly hair”, diverse patterns of striate and diffuse keratoderma changes, particularly in palmoplantar skin) and cardiac disease features such as in the classic “Naxos disease” type or the “Carvajal syndrome” subtype, first identified in the year 2000 in three Ecuadorian families, which may also include damages inboth ventricles (for an anthology see [42]). The specific dominant and recessive forms of the human diseases ascribed to mutations of genes encoding desmosomal proteins have been dealt with in several specific recent review articles [42, 97–102]. 

## 4. Adhering Junctions in the Specialized Cells of the Cardiac Conduction System

The specialized cells of the mammalian cardiac conduction system are connected by three different types of AJs [103]: desmosomes, which in certain cell regions occur in impressively high packing density, as well as AJs and CJs (Figures 5(b)–5(d)). The conductive cells of the ovine and bovine Purkinje fiber systems have been studied in special detail with respect to their nature as modified cardiomyocytes and to the various forms of junctions of which a major proportion is located at lateral cell-cell contact sites [103–105]. These findings have led to the hypothesis that the abundance of apparently “normal-looking” desmosomes in the conductive tissue might also—and perhaps primarily—be affected by the desmosomal protein mutations in cases of human ARVC/D (Table 2), as also suggested from the much higher conduction speed of these “cell fibers” (see, e.g., [103, 106, 107]). 

The various size classes of the desmosomal protein-rich junctions connecting conduction cells are presented by desmoplakin immunoelectron microscopy in Figures 5(b)–5(d), including some very small junctions(arrowheads in Figure 5(b)). Frequently, a number of individual desmin-containing intermediate filaments can be resolved at such junctions (e.g., Figure 5(c)), often revealing closely-parallel plaque associations (e.g., arrows in Figure 5(d)).

## 5. Desmosomal Plaque Proteins in Special Non-Desmosomal Adhering Junctions (*Coniunctiones* and *Complexus Adhaerentes*)

Proteins of the plakophilin-subfamily of *armadillo* proteins are constitutive, apparently necessary components of desmosomal plaques [13, 108–113]. Their special organizational role and architectonic importance has been demonstrated perhaps most convincingly in the case of plakophilin-2 by gene abrogation as well as siRNA-mediated mRNA reduction experiments [36, 37, 39–41]. Moreover, the functional importance of some plakophilins, in particular plakophilin-2, may extend beyond desmosomal plaques to gap junctions [37, 38] and into the interior of the cell, including certain cytoplasmic as well as nuclear complexes [114–116]. 

**Figure 6:**
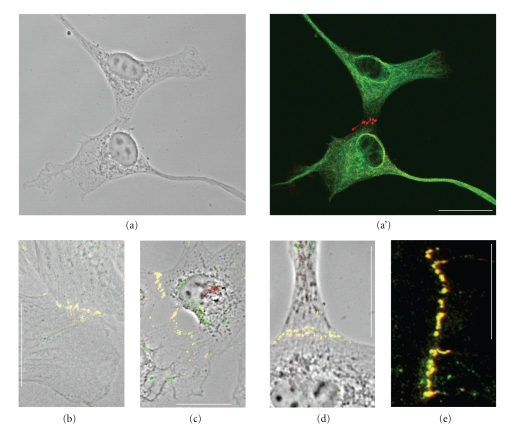
Double-label immunofluorescence micrographs, showing the localization of the “desmosomal protein”, plakophilin-2, in mesenchymally derived cultures of valvular interstitial cells (VICs). (a), (a′) Phase contrast and immunofluorescence micrograph showing ovine VICs forming clusters of AJs positive for the typical desmosomal plaque component plakophilin-2 (red; vimentin filaments are labeled in green). (b)–(d) Represent merged images of ovine VICs, showing colocalization staining (yellow) of plakophilin-2 (red) with typical AJ proteins such as cadherin-11 (green, (b)), *β*-catenin (green, (c)), protein p120 (green, (d)) all on a phase contrast background. (e) Human VICs exhibit similar colocalization of plakophilin-2 (red) here with N-cadherin (green) as shown by the yellow merge colour (for further details see [23]). Bar in (a): 25 *μ*m; bar in (b): 30 *μ*m; bars in (c) and (d): 20 *μ*m; bar in E: 10 *μ*m.

Recently, however, we have discovered that the occurrence of both plakophilin-2 and plakophilin-3 is not necessarily restricted to the plaques of desmosomes but that these proteins can also occur as constitutive molecules in plaques of some non-desmosomal junctions such as the *puncta adhaerentia*-like AJs of certain cell cultures [22, 23, 117] or in proliferatively active cells of certain tumors, for example in cardiac myxomata [118]. Here the rapid acquisition of plakophilin-2 to the non-desmosomal plaques of these tumor cell AJs, in particular the fact that it appears in the earliest in vitro culture passages of cardiac valvular interstitial cells from various mammalian species [23], suggests that the addition of plakophilin-2 alone—or together with plakophilin-3—to these junctions is somehow related to the induction of proliferation and cell cycle growth. It is therefore likely that in the future the systematic examination of the presence of plakophilins in such AJs will give valuable diagnostic informations. Figures 6(a)–6(e) present the early integration of plakophilin-2 into a series of small *puncta adhaerentia *connecting interstitial cells freshly brought from cardiac valve matrix tissue into cell culture (for details see [23]).

 The junctional system connecting the endothelial cells of blood and lymph vessels is obviously of great biological importance and has been studied extensively (reviews: [119, 120]). There is, however, a special category of variously-sized and -shaped AJs which connect certain types of endothelial cells in some parts of the lymphatic system, including the three-dimensionally branched “virgultar” cells of lymph node sinus as well as specific cutaneous and other peripheral lymph capillaries, which are characterized by AJs containing in addition desmoplakin as a major plaque protein, in most cases probably in combination with plakoglobin [18, 121–123]. The existence of such *complexus adhaerentes *in special parts of the vascular endothelial system has since been confirmed several times ([124–126], for a recent review see [19]). The formation of plaque complexes of VE-cadherin with desmoplakin and plakoglobin has also been demonstrated for dermal capillary endothelium in special molecular assembly experiments [127]. 

 Beyond this role of desmoplakin as a regular constituent of the plaques of such “complex junctions” in certain lymphatic endothelia of the mature body a fundamental and general role of desmoplakin in the formation of the vascular endothelial system is also indicated by the transgene embryogenesis studies of Gallicano et al. [128, 129]. Thus, the *complexus adhaerens *junctions have to be added to the list of novel kinds of adhering junctions in their own right (see Table 1; for recent reviews see: [19, 130, 131]).

## 6. Dispersed, Non-Junction Bound States of Desmosomal Cadherins: Desmoglein Dsg2

Desmosomal cadherins typically associate with each other and form close-packed cis-clusters in the membranes of cytoplasmic vesicles, then exocytose to form a “half-junction” on the cell surface and under sufficient Ca^2+^-concentration may further associate head-to-head in transform with another “half-junction”, usually a domain of an adjacent cell, to a symmetrical junction [132–137]. Isolated, that is, non-junction-bound, desmosomal cadherin molecules that have not been included in desmosome structures and consequently may be dispersed over extended parts of the plasma membrane until recently had not been observed in natural cells. Only in certain cell culture lines deficient of most junction components such as the human fibrosarcoma HT-1080 cells states of the isolated desmosomal cadherin, Dsg2, have been described to occur on cell surfaces and could be integrated into junction-like structures only upon introduction of further desmosomal proteins [137, 138]. 

Thus it was with great surprise when we noted the occurrence of Dsg2 molecules dispersed over large portions of the surface membrane of certain cultures of human melanocytes or neval cells as well as on surfaces of a subtype of melanoma cells in situ and in culture [139, 140]. As far as it could be concluded from the biochemical analyses and immunolocalization experiments so far performed, these Dsg2 glycoprotein molecules were not stably complexed with specific other transmembrane or with any plaque molecules but nevertheless seemed to be somehow involved in close membrane-to-membrane associations. It is obvious that such stages, that is, dispersed, non-junction-bound desmosomal cadherins will have to be studied with special care as they point to the existence of yet unknown, radically different cell-cell adhesion mechanisms involving desmosomal cadherins.

## Figures and Tables

**Table 1:** Shown are specific cell-cell adhering junctions containing transmembrane glycoproteins and cytoplasmic plaque proteins of “classic” desmosomes and adherens junctions (AJs). Special types of adhering junctions containing desmosomal proteins in human tissues and cell cultures.

Type	Cells	Transmembrane glycoproteins	Some representative plaque proteins	References
Macula adhaerens (desmosome)	Epithelial and mesothelial cells and carcinomas derived therefrom, cardiomyocytes of immature hearts and cardiac conductive cells	Desmogleins-1-4 Desmocollins-1-3	Plakophilins-1-3 Plakoglobin Desmoplakin	Franke et al. [10, 11] Cowin et al. [12] Mertens et al. [13]

Area composita (composite junction)	Cardiomyocytes and Purkinje fiber cells	N-Cadherin Cadherin-11 Desmoglein-2 Desmocollin-2	*α*- and *β*-Catenin Proteins p120, p0071 and ARVCF Plakoglobin Plakophilin-2 Desmoplakin Afadin Myozap	Franke et al. [14] Pieperhoff and Franke [15] Goossens et al. [16] Seeger et al. [17]

Complexus adhaerens	Certain endothelia (spec. endothelial and virgultar tissues of lymph node sinus)	VE-Cadherin N-Cadherin Claudin-5 JAM-A	*α*- and *β*-Catenin Protein p120 Plakoglobin Desmoplakin, Afadin	Schmelz and Franke Hämmerling et al. [18] Moll et al. [19]

Zona limitans externa	Neural retina	N-Cadherin	Neurojungin *α*- and *β*-Catenin Plakoglobin Plakophilin-2	Paffenholz et al. [20]

Colligatio permixta	Astrocytes and astrocytoma cells	N-Cadherin Cadherin-11 VE-Cadherin	*α*- and *β*-Catenin Protein p120 Plakoglobin Plakophilin-2 Afadin	Boda-Heggemann et al. [21]

Coniunctio adhaerens	Mesenchymally derived cells of high proliferative activity in situ and in culture	N-Cadherin Cadherin-11	*α*- and *β*-Catenin Proteins p120 and p0071 Plakoglobin Plakophilin-2 [Plakophilin-3] Afadin	Rickelt et al. [22] Barth et al. [23]

**Table 2:** During the last few years an avalanche of publications has appeared—and is still continuing to do so—showing the involvement of mutations in desmosomal components in the development of arrhythmogenic cardiomyopathies, including “sudden death” cases (for references see [10–12, 38, 40, 42–96]). Recent references reporting that certain mutations in human genes encoding desmosomal proteins and glycoproteins contribute to arrhythmogenic ventricular cardiomyopathies (ARVC).

Protein	Reference	
Plakophilin-2	Gerull et al. [43] Antoniades et al. [44] Calkins [45] Nagaoka et al. [46] Kannankeril et al. [47] Dalal et al. [48] Syrris et al. [49] Tsatsopoulou et al. [50] Van Tintelen et al. [51] Awad et al. [60] Lahtinen et al. [52] Otterspoor et al. [53]	Fidler et al. [38] Joshi-Mukherje et al. [54] Ram and Van Wagoner, [55] Tandri et al. [56] Wu et al. [57] Qiu et al. [58] (5 cases) Hall et al. [40] Bhuiyan et al. [59] (23 cases) den Haan et al. [64] (21 cases) Xu et al. [61] (38 cases) Bauce et al. [62] (7 cases) Cox et al. [95] (58 cases)

Desmoplakin	Norgett et al. [84] Rampazzo and Danieli [97] Alcalai et al. [86] Bauce et al. [77] Norman et al. [85] Sen-Chowdhry et al. [87] Norgett et al. [75] Uzumcu et al. [74] Sen-Chowdhry et al. [88]	Tsatsopoulou et al. [50] Yang et al. [89] den Haan et al. [64] (1 case) Mahoney et al. [81] Xu et al. [61] (10 cases) Bauce et al. [62] (5 cases) Cox et al. [95] (1 case) Bolling et al. [96]

Desmoglein-2	Pilichou et al. [90] Tsatsopoulou et al. [50] Awad et al. [60] Syrris et al. [73] Yu et al. [91]	Bhuiyan et al. [59] (4 cases) den Haan et al. [64] (8 cases) Xu et al. [61] (10 cases) Bauce et al. [62] (4 cases) Cox et al. [95] (3 cases)

Desmocollin-2	Heuser et al. [83] Syrris et al. [72] Beffagna et al. [79] Bhuiyan et al. [59] (2 cases)	Simpson et al. [76] Xu et al. [61] (4 cases) Bauce et al. [62] (2 cases) Cox et al. [95] (3 cases)

Plakoglobin	McKoy et al. [80] Protonotarios et al. [78, 82] Kaplan et al. [71] Garcia-Gras et al. [92]	Asimaki et al. [93] Asimaki et al. [94] den Haan et al. [64] (1 case) Xu et al. [61] (2 cases)

Selected review articles: Bazzi and Christiano [65]; Marcus et al. (Eds.) [42]; Awad et al. [66]; Corrado et al. [67]; Herren et al. [68]; Saffitz [69]; Sen-Chowdhry et al. [63].

First animal model (boxer dog): Oxford et al. [70].
